# Current-Induced Ductility Enhancement of a Magnesium Alloy AZ31 in Uniaxial Micro-Tension Below 373 K

**DOI:** 10.3390/ma12010111

**Published:** 2018-12-31

**Authors:** Xinwei Wang, Antonio J. Sánchez Egea, Jie Xu, Xianyu Meng, Zhenlong Wang, Debin Shan, Bin Guo, Jian Cao

**Affiliations:** 1 Laboratory for Space Environment and Physical Sciences, Harbin Institute of Technology, Harbin 150001, China; xinweiwang@hit.edu.cn; 2 School of Mechanical Engineering, Harbin Institute of Technology, Harbin 150001, China; wangzl@hit.edu.cn; 3 Department of Mechanical and Metallurgical Engineering, Pontificia Universidad Católica de Chile, Santiago 8320000, Chile; antonio.egea@ing.puc.cl; 4 Key Laboratory of Micro-Systems and Micro-Structures Manufacturing of Ministry of Education, Harbin Institute of Technology, Harbin 150080, China; xjhit@hit.edu.cn (J.X.); shandb@hit.edu.cn (D.S.); 5 School of Materials Science and Engineering, Harbin Institute of Technology, Harbin 150001, China; mengxianyu@outlook.com; 6 Department of Mechanical Engineering, Northwestern University, Evanston, IL 60208, USA; jcao@northwestern.edu

**Keywords:** ductility, fracture behavior, size effect, electrically assisted, micro-tension

## Abstract

The size effects in metal forming have been found to be crucial in micro-scale plastic deformation or micro-forming processes, which lead to attenuation of the material’s formability due to the increasing heterogeneity of the plastic flow. The use of an electric field during micro-scale plastic deformation has shown to relieve size effects, enhance the material’s formability, modify the microstructure, etc. Consequently, these electric-assisted (EA) micro-forming processes seem to bring many potential benefits that need to be investigated. Accordingly, here we investigated the influence of an electric field on the size effects to describe the fracture behavior in uniaxial micro-tension tests of an AZ31 alloy with various grain sizes. In order to decouple the thermal-mechanical and microstructure changes, room temperature (RT), oven-heated (OH), air-cooled (AC), and EA uniaxial micro-tension tests were conducted. The size effects contribution on the fracture stress and strain showed a similar trend in all the testing configurations. However, the smallest fracture stresses and the largest fracture strains were denoted in the EA configuration. EBSD examination shows that current-induced dynamic recrystallization (DRX) and texture evolution could be negligible under the studied conditions. The kernel average misorientation (KAM) maps give the larger plastic deformation in the EA specimens due to the reduction of plastic micro-heterogeneity. Finally, the fracture morphology indicates that the current-induced ductility enhancement may be attributed to the arrest of micro-crack propagation and the inhibition of void initiation, growth, and coalescence caused by lattice melting and expansion.

## 1. Introduction

For decades, micro-forming has been widely studied since it is a large-scale and green manufacturing technology for meso/micro scale components and assemblies. However, the so-called size effects have proven to influence on the material deformation behavior and tool performance, leading to a reduction of the forming limit and accuracy. These performance degradations would become more severe for magnesium alloys due to the hexagonal close packed (HCP) crystal structure. Thus, there are two possible methods to decrease these size effects: increase the forming temperature and refine the grain size. First, the increase of temperature will increase the possibility of tool breakage and surface oxidation. While the grain refinement will increase the forming loads and decrease the forming limit at room temperature (RT). Therefore, as a long-term vision, we attempt to find a novel process with the capability of controlling both the size effects and the tool life improvement in micro-forming. Significant efforts have been done to understand the electroplastic treatment to enhance the material’s formability in electric-assisted (EA) deformation. For example, it has been claimed that some direct interactive effects may exist between electrons and dislocations [1] during EA deformation. In particular, the electron wind [2] theory, based on the classical law of momentum transfer, has received a lot of attention. In our previous work [3], we stated that an electric field significantly affects the nature of grain boundaries via a localized Joule heating effect, which was experimentally proved by Zhan et al. [4]. The electromagnetic and thermal effects [5] were investigated in conductive materials in order to describe the stress distribution and the fracture area. They found that the Joule heating effect can induce local compressive thermoelastic stresses and melting areas at the crack tip which help to inhibit the crack propagation. Subsequently, these compressive stresses and crack inhibition would delay the fracture failure of the specimen. Based on these works, EA micro-forming may have the potential to improve the formability in micro-scale plastic deformation under lower operating temperatures and take less time. Also, the electric treatment seems to circumvent the size effects associated with traditional micro-forming in metals.

Until now, much literature [6,7,8] has been published about the electroplastic effect on different alloys and loading conditions, such as tensile, compressive, bending, and drawing. In general, the results tend to describe an enhanced formability, a reduced flow stress, and lower springback. Recent developments have focused on different bulk deformations assisted by an electric field. The potential benefit is to enhance the material formability and reduce the springback by promoting a fast microstructure alteration, and subsequently, a high-rate transformation of mechanical properties. Accordingly, Kuang et al. [9] reported the influence of temperature using furnace and electropulsing treatments when rolling a Mg alloy. They found significant differences in the recrystallization mechanism and material texture. These changes were attributed to the magnification of dynamic recrystallization (DRX) because the electric field strongly induced a micro-shear band when low accumulative true strains were assessed. In addition, Sánchez Egea et al. [10] described the X-ray pattern of drawn 308L specimens assisted by electropulses. They stated that the electropulses promoted an ultra-fast annealing treatment in the specimen’s microstructure. This annealing treatment could explain the formability enhancement in these EA wire-drawn specimens. Similarly, Xie et al. [11] denoted a detwinning mechanism in Mg strips after an EA bending process. A dissimilar misorientation angle distributions of the detwinning mechanism was found when comparing the microstructure of as-received, conventional, and EA-bent specimens. Recently, the fracture mechanism in Ti6Al4V sheet metals that underwent an EA double-sided incremental forming technique was characterized by Valoppi et al. [12]. They described that electric current has a localized thermal softening associated with it that helps to enhance the shear effect and reduce the surface damage.

Regarding these works, the effort to relieve the size effects by using an electric field has become a hot topic in micro-forming processes. Previous works have proposed different models to describe the influences of an electric field in microtension, i.e. the Hall-Petch effect [3], the size effect on softening behavior [13], and the thermal and flow behaviors [14]. However, very limited results have been reported to denote the importance of the fracture behavior in EA micro-formed specimens. In this study, we aim to investigate the size effects on the fracture behavior during a tensile test under different configurations: RT, oven-heated (OH), EA, and air-cooled (AC). Subsequently, the microstructure evolution and fracture analysis are provided to analyze the current-induced ductility enhancement and describe a possible fracture mechanism in EA micro-tension.

## 2. Experiment

**Figure 1:**
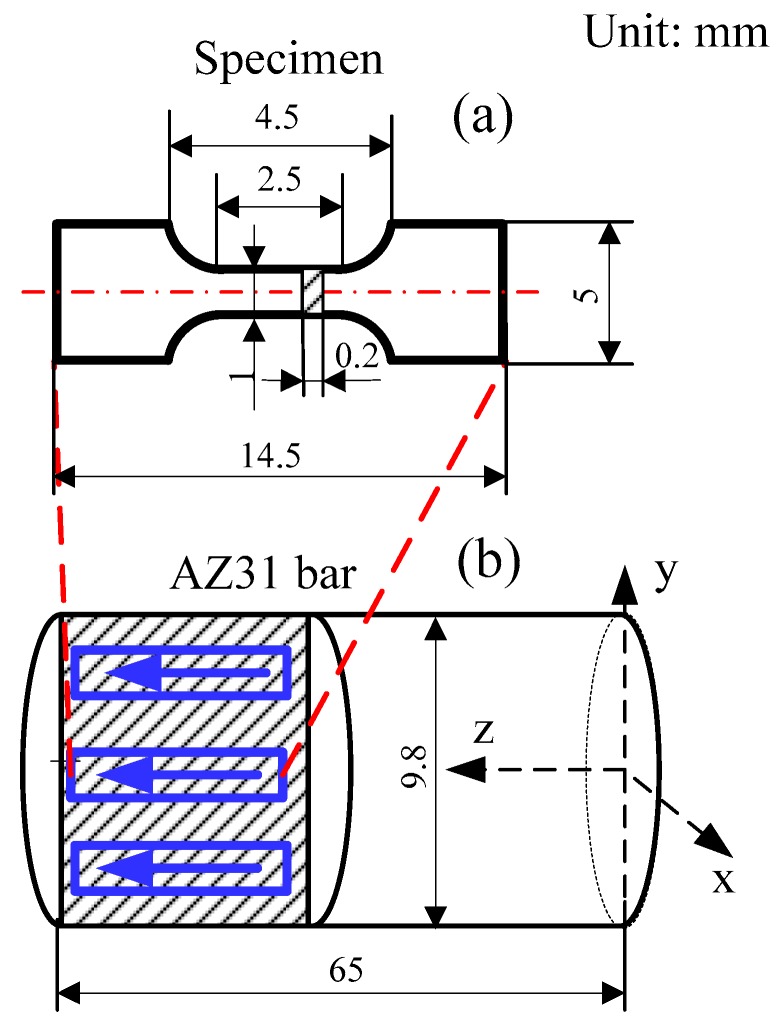
(**a**) Dimensions of the micro-tension specimens, and (**b**) illustration of the dog-bone shape specimen preparation.

Uniaxial micro-tension tests were performed on AZ31 magnesium alloy (94.8% Mg, 3.5% Al, 1.2% Zn and 0.5% Mn, MTI Instruments, Inc, NY, USA) specimens. Dog-bone shaped specimens were done for the uniaxial micro-tension, as shown in Figure 1. Furthermore, all the specimens were annealed with the same protocol as in our previous work [3]. Accordingly, four different grain sizes were obtained before the micro-tension tests, i.e., 7.1 ± 1.1 μm, 17.6 ± 2.3 μm, 43.3 ± 4.4 μm, and 99.8 ± 24.9 μm.

Micro-tension specimens were conducted on a commercial scanning electron microscope (SEM, Core Electron Microscopy Facility, MA, USA) loading stage. A continuous electric current was supplied by using a rectifier-based DC power supply with 3600 W peak power. A load cell with a working range of 100 N and a resolution of 0.01 N was used to measure the micro-tensile force. The displacement and strain were calculated using a 2-D digital image correlation (DIC) method, based on the patterned images captured by a high-resolution CCD camera (Edmund Optics TECHSPEC Silver Series, NJ, USA). Moreover, an infrared camera was used to record the temperature during the experiments. To decouple thermal-mechanical and other microstructure effects caused by the electric field, both OH and AC configurations were conducted with similar bulk temperatures as the EA and RT configurations, respectively. The detailed setup configurations and testing procedures were given in our previous work [3]. 

Electron backscattered diffraction (EBSD) examinations were conducted at the fracture area of samples subjected to the RT, OH, AC, and EA micro-tension tests. In particular, a Quanta 200FEG field emission scanning electron microscopy (FESEM) device (Core Electron Microscopy Facility, MA, USA) with a working distance of ≈13 mm was used to record the EBSD. The EBSD data were analyzed with a orientation imaging microscopy (OIM) software (OIM Analysi v8, Edax, Beijing, China) provided with the SEM. For each configuration, one fractured sample was randomly chosen to analyze the microstructure. All the samples were grouped and mounted with epoxy, ground by hand under flowing water using 320-grit, 400-grit, and 800-grit sandpapers, and finally, a fine-ground sandpaper of 1200-grit. Later, the samples were polished with 5 μm, and later, 0.05 μm alumina powder on a polishing wheel under flowing water. Afterwards, the mechanically-polished specimens were electro-polished using a solution of 37.5% H_3_PO_4_ and 62.5% C_2_H_5_OH under a DC voltage of 5 V at ≈293 K until a mirror-like surface was achieved. Finally, the electro-polished samples were rinsed in methyl alcohol and ethanol alcohol consecutively. The fracture area of each specimen configuration was observed using the aforementioned electron microscopy devices.

## 3. Results and Discussion

**Figure 2:**
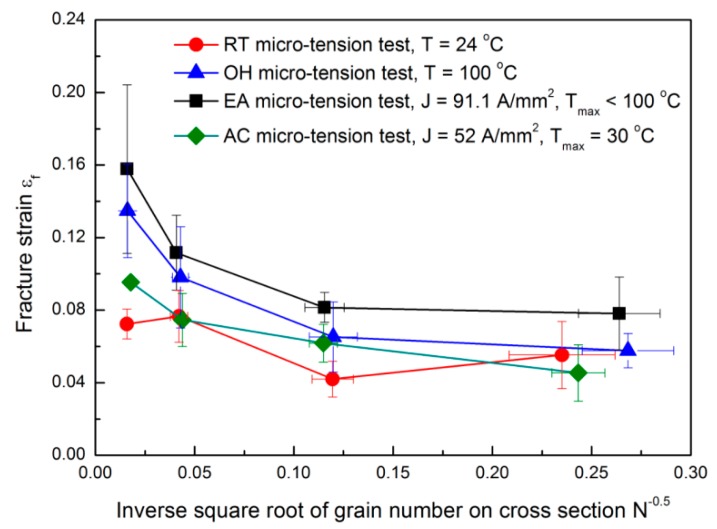
The fracture strain behavior for the different tested configurations.

**Figure 3:**
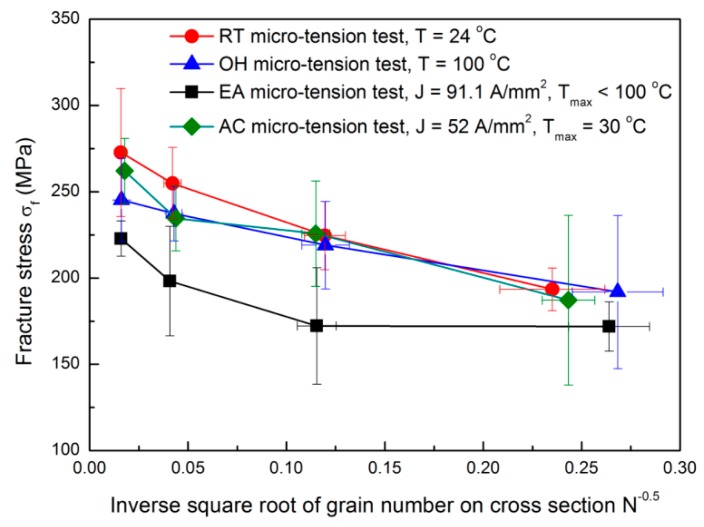
The fracture stress behavior for the different tested configurations.

The size effects on fracture behavior depends on the dislocation density and its distribution in the material’s microstructure. These two parameters can be roughly indicated by the fraction of grain boundaries, since the dislocation density at the grain boundaries are typically found in a high concentration [15]. A linear relationship between the grain boundary fraction and the reciprocal square root of the number of grains is expected. Also, it can be assumed to be a polycrystalline material with two phases, i.e., grain boundary network and interior grain. Subsequently, the size effects on fracture strain and stress can be approximately characterized by the reciprocal square root of the number of grains. Figure 2 and Figure 3 show the fracture strain $\varepsilon_{f}$ and stress $\sigma_{f}$ variations with respect to the inverse square root of grain number found in the sample cross sectional area. *N* is quantified with the ratio of the specimen cross sectional area with respect to the grain cross sectional area. For all the cases, the fracture strain and stress increase when increasing *N*; similar results were found by Fu and Chan [15]. The increase of fracture stress with the increase of *N* is associated with the grain boundary strengthening effect because the grain boundary fraction also increases with *N* and the dislocation motion is mainly impeded by the grain boundaries. Accordingly, the EA and RT configurations present the smallest and the largest fracture stresses, respectively. While, the fracture stresses do not show significant differences among the RT, OH, and AC configurations when a smaller *N* is denoted. Consequently, for a low *N*, the fracture behavior is expected to be dominated by few grains with preferential orientations, locations and sizes, causing a relatively larger uncertainty of fracture stress [16]. Meanwhile, for a high *N*, the current-induced effect on fracture behavior may outweigh the influence of microstructure deviation, and as a consequence, reducing the fracture stress in the following order: RT, AC, OH, and EA micro-tension configurations.

Micro-voids are likely to nucleate at the grain boundaries, where the stress concentration shows a noticeable increase due to the strain incompatibility of neighboring grains [17]. Hence, we could expect that a localized thermal heating in the micro-voids’ surroundings and grain boundaries may occur under the EA configurations. This localized thermal heating at the grain boundaries was further discussed in our previous works [3,10]. Thus, if the grain boundary density increases with *N*, more micro-voids would be heated by the localized thermal effects causing a higher reduction of the fracture stress.

Figure 2 shows a decrease of the fracture strain associated with a decrease of *N*. This could be also attributed to an inhomogeneous deformation and anisotropic stress concentrations. Specifically, when the grain size approaches to the specimen size (low *N*), the behavior of the plastic deformation would be dominated by the properties (i.e., location, size, and orientation) of a few preferentially oriented grains. In this particular case, only a few slip systems were activated causing severe strain incompatibility and an early fracture. Looking at Figure 2, the EA micro-tension configuration denotes the highest ductility. This result is contrary to the one reported by Ross et al. [18]. They found that a continuous electric field reduced the maximum achievable elongation of a metal under a tensile test. The key parameter is the bulk temperature, which in our EA configuration, achieved a lower temperature of about 373 K. This temperature can cause a smaller strain localization, and consequently, more delayed current-induced failure in a localized area. However, excessive heating and severe necking occurred in the round specimens with a larger gauge length and diameter. This excessive temperature will eventually contribute to an early fracture failure, as reported in [18]. Note that the fracture strain in the OH specimen is smaller than the EA specimen, although the temperature in the OH specimen (i.e., 373 K) is higher than the EA specimen. The same happens if AC and RT configurations are compared, where AC shows a slightly higher fracture strain when increasing N. Consequently, we could assume that the influence of the electric field on ductility could not be considered to be exclusive to the bulk heating. The electric field also affected the stress and strain concentration and dislocation movement on the material’s microstructure in terms of the nucleation, growth, and coalescence of micro-voids. For these reasons, the current-induced ductility enhancement was observed to be more significant with higher *N*, although more micro-voids would be expected to form at the grain boundaries. As a result, the electric field may have the ability to impede the growth and coalescence of micro-voids, which can inhibit the crack propagation, as also stated by Liu [5].

**Figure 4:**
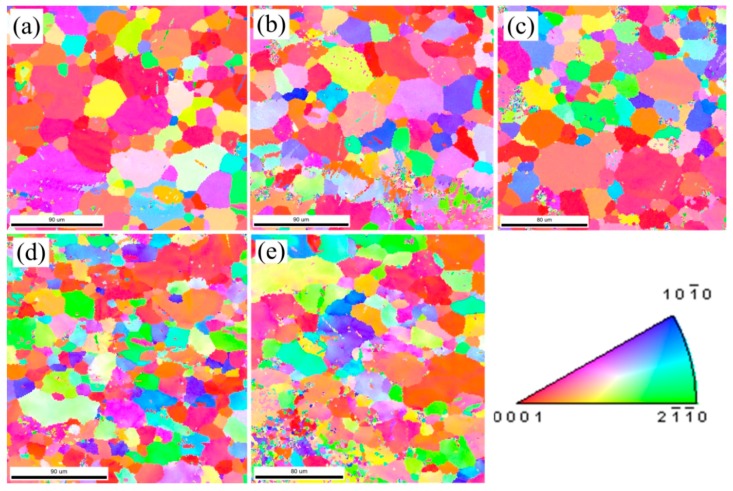
Colored IPF maps obtained from EBSD analysis in ND near the fracture areas of the failure samples for the different tested configurations: (**a**) undeformed material, (**b**) RT, (**c**) OH, (**d**) AC, and (**e**) EA and the color pallet associated to the Miller indices.

**Figure 5:**
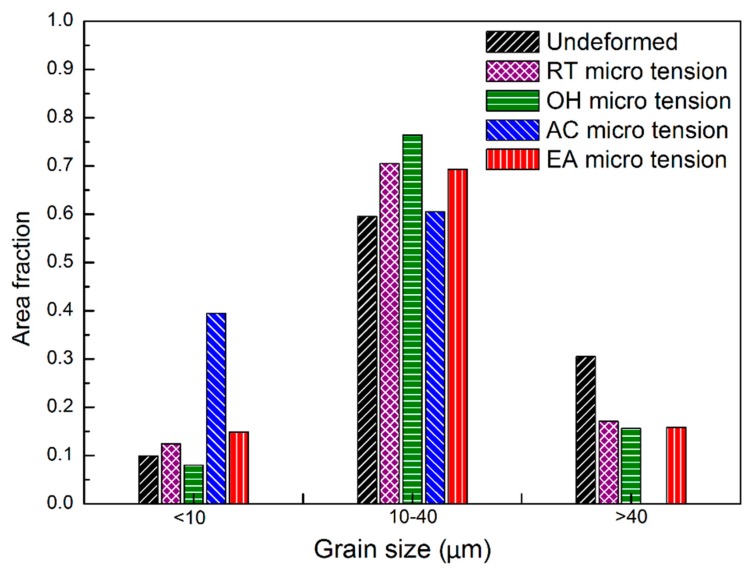
Grain size distributions of the fractured AZ31 samples for the different tested configurations.

**Figure 6:**
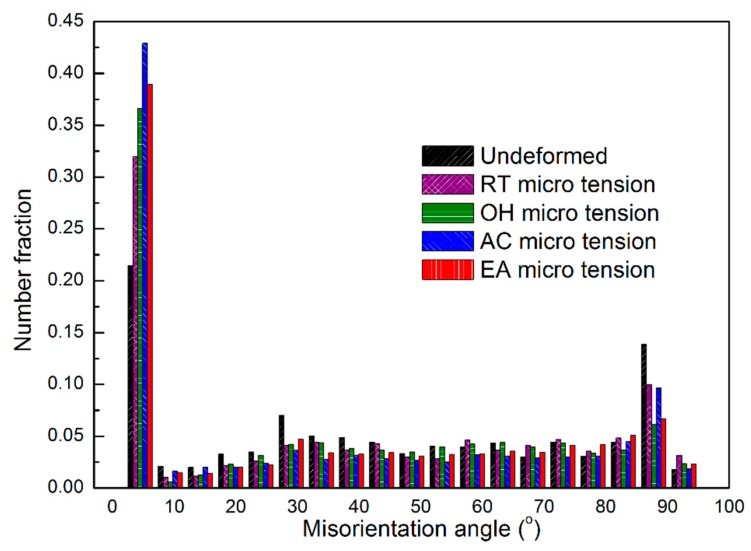
Pixel-to-pixel misorientation angle distributions of the fractured AZ31 samples for the different tested configurations.

Figure 4 shows the inverse pole figure (IPF) maps in the normal direction (ND) at the fracture area for the different tested configurations. The initial microstructure (Figure 4a) has relatively equiaxed grains with a grain size of about 20 μm. The same size is inherited in Figure 4b, due to the small tensile deformation in the RT configuration. The microstructure in the OH specimen (Figure 4c) is approximately consistent with the annealed material. There was no evidence of grain growth, so DRX did not occur in these samples, where the recorded temperature was 373 K, far below the DRX temperature in AZ31 [19]. However, more fractions of small grains (i.e., <10 μm) were observed in the fractured samples subjected to an electric current (as shown in Figure 4d,e), especially for the AC configuration. This could be associated to a localized thermal heating that reached an instantaneous high temperature to cause grain nucleation at the microstructure defects and/or inclusions. Comparing the initial grain size distribution with all the deformed samples under different micro-tension conditions, a lower fraction of >40 μm grains is denoted. On the contrary, Figure 5 shows a similar grain fraction within the range of 10–40 μm. From these results, a dynamic grain growth was negligible, even for the EA specimens, despite the fact that the opposite results were reported by Kim et al. [20]. We noted that EA specimens seemed not to noticeably change the grain size distribution compared to the RT and OH configurations. In this study, DRX could not be seen as the key factor causing the ductility enhancement and current-induced softening for two reasons: the test duration (<50 s) here was not as long as in Reference [20] to cause grain growth, and the Joule heating effect was not high enough to reach the DRX temperature of AZ31. From another point of view, however, the temperature gradient, due to electric resistance heating, could provide enough driving force for grain nucleation and growth. In this study, premature fracture terminated the subsequent growth of the nucleated grains, leading to higher concentrations of small grains (<10 μm) spreading along the grain boundaries in the AC and EA configurations. Figure 6 shows the pixel-to-pixel misorientation angle distributions with local maxima at ≈5°. Accordingly, the fraction value of low-angle misorientations (i.e., <15° pixel-to-pixel misorientation) increased in the following order: the undeformed material, RT, OH, EA, and AC, respectively. For example, over 40% low-angle misorientations could be observed in EA and AC configurations. Consequently, the EA specimens promoted the formation of low misorientation angles while being deformed with respect to the RT, OH, and the undeformed material.

**Figure 7:**
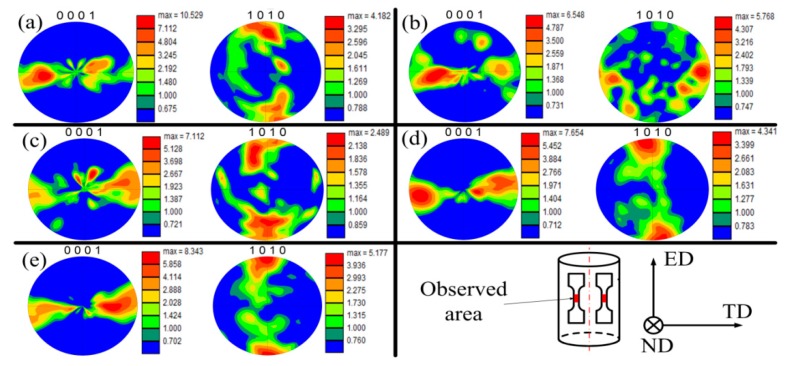
Texture evolution of the {0001} and {10$\bar{1}$0} in fractured AZ31 samples in the ND plane for the different tested configurations: (**a**) undeformed material, (**b**) RT, (**c**) OH, (**d**) AC, and (**e**) EA and the sample orientation respect to the main directions.

Figure 7 presents the pole figures (PFs) with the aim of explaining the texture evolution of the current-induced effect. All the samples were cut from the axial cross section of extrusion bars along extrusion direction (ED) (Figure 1). Consequently, a typical texture for extruded AZ31 [21] was found where the c-axis was distributed perpendicular to the ED. The texture found was inherited from the annealed material (Figure 7a) that was previously extruded. Figure 7b shows a basal pole density maximum that moves towards the ED, while the {10$\bar{1}$0} pole tends to reorient from ED to transverse direction (TD). These observations indicate that both twinning and prismatic <a>-type slips occurred in the RT specimen. The twins tended to reorient the basal pole towards ED [22] and the prismatic slip tended to rotate the crystallites around the c-axis without changing its direction [23]. Figure 7c shows the PF of the OH specimen. Here the twinning activity was not as remarkable as in the RT, perhaps because certain non-basal slips could be activated to accommodate the plastic deformation. The temperature favored this accommodation by decreasing the critical resolve shear stresses (CRSS). Additionally, Figure 7d,e exhibits the PFs of the AC and EA samples, respectively. In these cases, electric treatments show a similar texture to the undeformed material where the {0001} and {10$\bar{1}$0} poles tend to be parallel to the TD and ED, respectively. 

Moreover, the electric treatment does not promote obvious twinning activities. The preservation of the initial texture after ≈15% of plastic strain in the EA configuration indicated that not only grain rotation but also other factors gave rise to the texture stabilization. It is considered that grain boundary sliding (GBS) combined with grain rotation occurred in the EA sample to decrease the texture intensity [24] compared to the undeformed material, as observed in Figure 7e. The current-induced heating and the associated thermal softening at the grain boundaries [25] may have improved the pure GBS between neighboring grains. Accordingly, Koike et al. [26] pointed out that at temperatures below 373 K, combined with pure GBS, would help slip-induced GBS because of the plastic anisotropy and deformation inhomogeneity in the grains. Consequently, a possible deformation mechanism for the EA configuration is that both grain rotation in sliding grains and dislocation slip in accommodating grains (acting as an accommodation mechanism for GBS) may take place. These two mechanisms help each other in terms of changing the grain orientation. These orientation changes collectively lead to the stabilization and weakening of the initial texture, as illustrated by Watanabe et al. [24]. 

**Figure 8:**
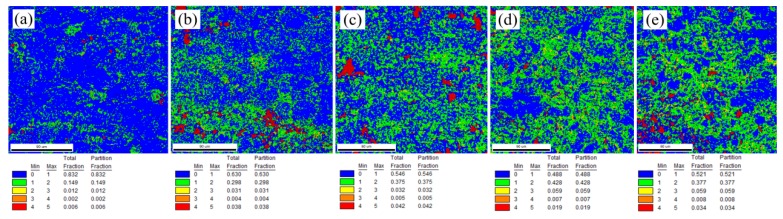
KAM map (in ^o^) of the fractured AZ31 samples for the different tested configurations: (**a**) undeformed material, (**b**) RT, (**c**) OH, (**d**) AC, and (**e**) EA (red points are artificial points).

The KAM is defined as the average misorientation of a given EBSD point with respect to all its neighbors, which is usually used to characterize the distribution of the geometrically necessary dislocation (GND) density [27]. Figure 8a–e shows the KAM maps of all the studied microtension configurations. No obvious misorientation gradient was expected, and almost all the KAM values were less than 2° (having a total fraction of 0.981) prior to the deformation (Figure 8a). Figure 8b exhibits the tensile deformation at RT. Here the misorientation gradient obviously increased, e.g., the fractions of <2° and 2°–4° misorientations are 0.928 and 0.035, respectively. This is attributed to the dislocation accumulation and multiplication caused by the local inhomogeneous deformation of an anisotropic plastic behavior. Additionally, Figure 8c shows the OH misorientation gradient, where the fractions of <2° and 2°–4° misorientations are 0.921 and 0.037, respectively. These changes as compared to the RT sample, present a larger plastic deformation associated with the higher temperature applied in OH. Note also that the KAM distribution in Figure 8c is more uniform because the furnace treatment increased the temperature uniformly, which helped the activation of multiple slip activities in unfavorably oriented grains. Figure 8d,e exhibits the KAM maps of AC and EA specimens, respectively. Their GNDs were more dense and more uniform compared to the OH sample. As well, the fractions of the large KAM (2°–4°) were 1.5 times the value of the fraction of OH. This uniform GND distribution indicates that more grains took part in plastic deformation to a larger degree, enhancing the ductility of the EA sample. Accordingly, the electric thermal heating tended to occur at the dislocations, not only in grain interiors, but also between neighboring grains, which facilitated the dislocation mobility if sufficient energy was supplied to the material’s lattice. As a result, the plastic accommodation among the vicinities of different defects in the grain interiors and grain boundaries could be easily accomplished by enhancing local activation of dislocations. This activation was induced by the selective electric thermal heating at the microstructure defects, inclusions, or vacancies. Subsequently, it was presumed that there may exist a current induced effect to eliminate the micro-heterogeneity, and ultimately, relieve the size effects in microforming [16].

**Figure 9:**
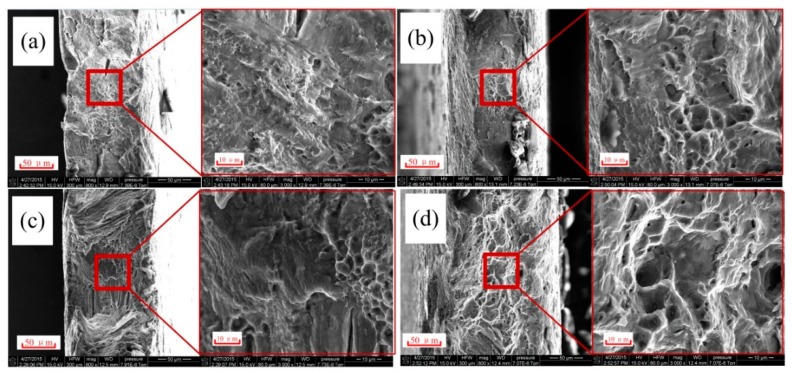
SEM micrographs of the fracture areas for (**a**) RT, (**b**) OH, (**c**) AC, and (**d**) EA.

Figure 9 shows the SEM micrographs of the fracture area of different studied configurations. This figure let us characterize the fracture mechanism by analyzing the surface of the final fracture area. Figure 9a shows the fracture area for RT. This sample presented the features of a cleavage fracture, which is composed of tear ridges and large quasi-cleavage facets and steps. Also, some large secondary cracks could be found perpendicular to the main crack. Figure 9c exhibits the fracture area of AC, which contains large and rough tear ridges and a high number of cleavage facets and steps. A few small and shallow dimples can also be found, indicating that this specimen presents higher ductility than RT. Figure 9b presents the fracture area of OH with the same cleavage features and large cleavage facets. This specimen shows a higher number of dimples with deeper and larger dimensions than in AC, denoting a higher plastic deformation. Figure 9d describes the fracture of EA. This fracture morphology presents the large dimple patterns associated with a ductile fracture. These dimples are the deepest and largest compared to other configurations. In general, dimple formation originate from the coalescence of voids with different sizes. These voids tend to form along the interface of embedded particles/inclusions during tensile loading [28]. In this study, we could neglect the influence of material-dependent inclusions on the dimple size difference because all the samples of different configurations were machined from the same extruded bar. Thus, certain current-induced effects (probably different from convention heating) may exist to affect the dimple size and fracture behavior in the EA configuration. 

**Figure 10:**
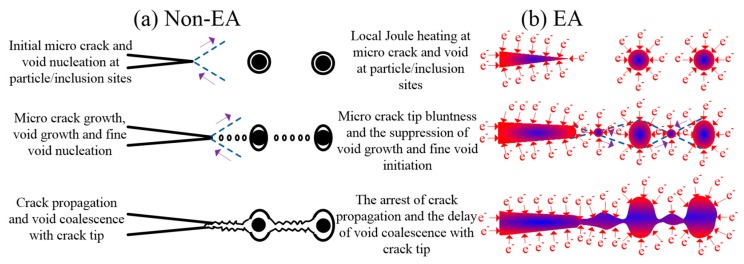
Expected mechanism of crack propagation, void nucleation, growth and coalescence: (**a**) non-EA and (**b**) EA configurations.

Figure 10 describes the expected fracture mechanisms for the non-EA and EA configurations. The fracture process of AZ31 magnesium alloy was assumed to mainly depend on the interaction between microcracks and voids (Figure 10a). They can be nucleated near particles/inclusions or their interfaces due to stress concentration or poor bonding strength at a low plastic strain. Additionally, a large number of small voids may easily nucleate between growing voids and cracks. Consequently, the fracture will appear with the coalescence of voids, particle/inclusions, and micro-cracks along the weakest path of the material’s lattice [29]. Figure 10b shows the expected fracture mechanism for an EA configuration. Here, an intense diffraction of electrons is shown around the microstructure’s defects, such as the interfaces of micro-cracks, inclusions, particles, etc. These defects would promote hot spots due to the fast change of resistivity. Accordingly, many researchers [30,31] claimed that the current-induced heating effect in the material’s lattice would reduce stress concentration and increase thermo-compressive stress around microstructural defects such as micro-crack tips, voids, etc. This may be supported by the fatigue lifetime improvement of AISI 304 due to high density electropulsing observed by Lesiuk et al. [32]. Thus, the ductility improvement in the EA samples may be attributed to the following two aspects: (1) the electric field tends to arrest the propagation of micro-cracks, and (2) the electric field has the ability to suppress the initiation of fine voids and impede the growth and coalescence of existing voids. 

## 4. Conclusions

The size effects on the fracture behavior of magnesium alloy AZ31 in uniaxial micro-tension under different temperature configurations have been provided several notable conclusions. The following bullets summarize these conclusions:

- The material’s formability changed depending on the thermal configuration (EA, OH, or AC). In particular, the smallest fracture stresses and the largest fracture strains were observed in the EA microtension configurations.
- The EBSD analysis showed a similar grain size distribution in all the microtension configurations, indicating that DRX did not play a key role in the ductility enhancement of EA samples.
- The KAM characterization denoted that plastic heterogeneity could be reduced since more grains took part in plastic deformation in EA specimens, and consequently, led to the ductility enhancement.
- The fracture morphology observed in the SEM micrographs indicated that the current-induced ductility enhancement could be attributed to the arrest of microcrack propagation and the inhibition of void initiation, growth, and coalescence caused by lattice melting and expansion.

## References

[1] Guan L., Tang G., Chu P.K. (2010). Recent advances and challenges in electroplastic manufacturing processing of metals. J. Mater. Res..

[2] Sprecher A., Mannan S., Conrad H. (1986). Overview No. 49: On the mechanisms for the electroplastic effect in metals. Acta Metall..

[3] Wang X., Xu J., Jiang Z., Zhu W.-L., Shan D., Guo B., Cao J. (2016). Size effects on flow stress behavior during electrically-assisted micro-tension in a magnesium alloy AZ31. Mater. Sci. Eng. A.

[4] Zhang X., Li H., Zhan M. (2018). Mechanism for the macro and micro behaviors of the Ni-based superalloy during electrically-assisted tension: Local Joule heating effect. J. Alloys Compd..

[5] Liu T.J.C. (2011). Finite element modeling of melting crack tip under thermo-electric Joule heating. Eng. Fract. Mech..

[6] Nguyen-Tran H.-D., Oh H.-S., Hong S.-T., Han H.N., Cao J., Ahn S.-H., Chun D.-M. (2015). A review of electrically-assisted manufacturing. Int. J. Precis. Eng. Manuf.-Green Technol..

[7] Salandro W.A., Jones J.J., Bunget C., Mears L., Roth J.T. (2015). Electrically Assisted Forming: Modeling and Control.

[8] Egea A.J.S., González-Rojas H.A., Celentano D.J., Peiró J.J., Cao J. (2017). Thermomechanical Analysis of an Electrically Assisted Wire Drawing Process. J. Manuf. Sci. Eng..

[9] Kuang J., Du X., Li X., Yang Y., Luo A.A., Tang G. (2016). Athermal influence of pulsed electric current on the twinning behavior of Mg–3Al–1Zn alloy during rolling. Scr. Mater..

[10] Egea A.J.S., Rojas H.A.G., Celentano D.J., Peiró J.J. (2016). Mechanical and metallurgical changes on 308L wires drawn by electropulses. Mater. Des..

[11] Xie H., Dong X., Peng F., Wang Q., Liu K., Wang X., Chen F. (2016). Investigation on the electrically-assisted stress relaxation of AZ31B magnesium alloy sheet. J. Mater. Process. Technol..

[12] Valoppi B., Zhang Z., Deng M., Ghiotti A., Bruschi S., Ehmann K.F., Cao J. (2017). On the Fracture Characterization in Double-Sided Incremental Forming of Ti6Al4V Sheets at Elevated Temperatures. Procedia Manuf..

[13] Wang X., Xu J., Shan D., Guo B., Cao J. (2017). Effects of specimen and grain size on electrically-induced softening behavior in uniaxial micro-tension of AZ31 magnesium alloy: Experiment and modeling. Mater. Des..

[14] Wang X., Xu J., Shan D., Guo B., Cao J. (2016). Modeling of thermal and mechanical behavior of a magnesium alloy AZ31 during electrically-assisted micro-tension. Int. J. Plast..

[15] Fu M., Chan W. (2011). Geometry and grain size effects on the fracture behavior of sheet metal in micro-scale plastic deformation. Mater. Des..

[16] Fu M.W., Wang J.L., Korsunsky A.M. (2016). A review of geometrical and microstructural size effects in micro-scale deformation processing of metallic alloy components. Int. J. Mach. Tools Manuf..

[17] Querin J.A., Schneider J.A., Horstemeyer M.F. (2007). Analysis of micro void formation at grain boundary triple points in monotonically strained AA6022-T43 sheet metal. Mater. Sci. Eng. A.

[18] Ross C.D., Irvin D.B., Roth J.T. (2007). Manufacturing aspects relating to the effects of direct current on the tensile properties of metals. J. Eng. Mater. Technol..

[19] Tan J., Tan M. (2003). Dynamic continuous recrystallization characteristics in two stage deformation of Mg–3Al–1Zn alloy sheet. Mater. Sci. Eng. A.

[20] Kim M.-J., Lee K., Oh K.H., Choi I.-S., Yu H.-H., Hong S.-T., Han H.N. (2014). Electric current-induced annealing during uniaxial tension of aluminum alloy. Scr. Mater..

[21] Barnett M. (2007). Twinning and the ductility of magnesium alloys: Part I: “Tension” twins. Mater. Sci. Eng. A.

[22] Koike J. (2005). Enhanced deformation mechanisms by anisotropic plasticity in polycrystalline Mg alloys at room temperature. Metall. Mater. Trans. A.

[23] Valle J.A.D., Pérez-Prado M.T., Ruano O.A. (2005). Deformation mechanisms responsible for the high ductility in a Mg AZ31 alloy analyzed by electron backscattered diffraction. Metall. Mater. Trans. A.

[24] Watanabe H., Kurimoto K., Uesugi T., Takigawa Y., Higashi K. (2013). Accommodation mechanisms for grain boundary sliding as inferred from texture evolution during superplastic deformation. Philos. Mag..

[25] Fan R., Magargee J., Hu P., Cao J. (2013). Influence of grain size and grain boundaries on the thermal and mechanical behavior of 70/30 brass under electrically-assisted deformation. Mater. Sci. Eng. A.

[26] Koike J., Ohyama R., Kobayashi T., Suzuki M., Maruyama K. (2005). Grain-Boundary Sliding in AZ31 Magnesium Alloys at Room Temperature to 523 K. Mater. Trans..

[27] Dutta R.K., Petrov R.H., Delhez R., Hermans M.J.M., Richardson I.M., Böttger A.J. (2013). The effect of tensile deformation by in situ ultrasonic treatment on the microstructure of low-carbon steel. Acta Mater..

[28] Shih T.S., Liu W.S., Chen Y.J. (2002). Fatigue of as-extruded AZ61A magnesium alloy. Mater. Sci. Eng. A.

[29] Somekawa H., Mukai T. (2005). Effect of texture on fracture toughness in extruded AZ31 magnesium alloy. Scr. Mater..

[30] Sheng Y., Hua Y., Wang X., Zhao X., Chen L., Zhou H., Wang J., Berndt C.C., Li W. (2018). Application of high-density electropulsing to improve the performance of metallic materials: Mechanisms, microstructure and properties. Materials.

[31] Hameed S., Rojas H.A.G., Benavides J.I.P., Alberro A.N., Egea A.J.S. (2018). Influence of the Regime of Electropulsing-Assisted Machining on the Plastic Deformation of the Layer Being Cut. Materials.

[32] Lesiuk G., Zimniak Z., Wiśniewski W., Correia J.A.F.O. (2017). Fatigue lifetime improvement in AISI 304 stainless steel due to high-density electropulsing. Procedia Struct. Integr..

